# A DFT study of permanganate oxidation of toluene and its *ortho*-nitroderivatives

**DOI:** 10.1007/s00894-014-2091-1

**Published:** 2014-02-14

**Authors:** Paweł Adamczyk, Reto S. Wijker, Thomas B. Hofstetter, Piotr Paneth

**Affiliations:** 1 Institute of Applied Radiation Chemistry, Faculty of Chemistry, Lodz University of Technology, Zeromskiego 116, 90-924 Lodz, Poland; 2 Eawag, Swiss Federal Institute of Aquatic Science and Technology, 8600 Dübendorf, Switzerland

**Keywords:** Permanganate, B3LYP, DFT, HOMA, Nitroaromatic pollutants, Toluene

## Abstract

Calculations of alternative oxidation pathways of toluene and its *ortho*-substituted nitro derivatives by permanganate anion have been performed. The competition between methyl group and ring oxidation has been addressed. Acceptable results have been obtained using IEFPCM/B3LYP/6-31+G(d,p) calculations with zero-point (ZPC) and thermal corrections, as validated by comparison with the experimental data. It has been shown that ring oxidation reactions proceed via relatively early transition states that become quite unsymmetrical for reactions involving *ortho*-nitrosubstituted derivatives. Transition states for the hydrogen atom abstraction reactions, on the other hand, are late. All favored reactions are characterized by the Gibbs free energy of activation, ΔG^≠^, of about 25 kcal mol^−1^. Methyl group oxidations are exothermic by about 20 kcal mol^−1^ while ring oxidations are around thermoneutrality.

**Figure:**
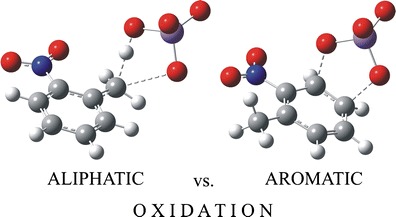
Oxidation of toluene and its ortho-nitroderivatives

Oxidation of toluene and its ortho-nitroderivatives

## Introduction

Anthropogenic influence on the natural environment results in the presence of a wide range of aromatic pollutants in soil, sediments, as well as surface- and groundwaters since aromatic compounds are widely used by industries but also they are a component of gasoline and oils [1, 2]. These compounds are of high toxicity, stability and ability of bioaccumulation and depending on the component of the ecosystem in which they are present, they may undergo a transition through the various abiotic or biological processes. In addition, products of such degradation reactions may also pose a significant environmental hazard [1, 2]. In recent years mechanisms of these processes have been intensively studied in search of the best methods for removal of aromatic compounds from the environment.

It has been shown than oxidative degradation of many of these contaminants, both biotic and abiotic, may proceed via two competitive pathways: aromatic ring oxidation and methyl group oxidation [3, 4]. In environmental field studies compound specific isotope analysis (CSIA) is increasingly used for quantitative estimates of ongoing degradation processes. In the case of polynitroaromatic pollutants, such as mono-, dinitrotoluenes, typical analysis of carbon and hydrogen isotope fractionation combined with reaction progress is difficult to establish as outlined in a companion paper [5]. Thus a more fundamental understanding of possible oxidation pathways is essential not only for the selection of an appropriate treatment but also for improvement of CSIA-based accessing of degradation processes of nitroaromatic compounds [6]. As permanganate, the most popular oxidant for the in situ chemical oxidation, is capable of oxidizing both aromatic ring and aliphatic chains [5, 7] we have used it as a model oxidant for our studies of oxidative degradation of common aromatic pollutants.

The mechanism of toluene oxidation by permanganate has been the subject of detailed experimental [8–12] and theoretical studies [13]. However degradation of nitroaromatic compounds by permanganate was not studied to an extent that would allow one to assess the relative shares of oxidation at the alkyl vs. aryl moieties. Herein we present detailed theoretical study of the rate-determining step of permanganate oxidation of three aromatic pollutants; toluene and its two nitro derivatives, 2-nitrotoluene and 2,6-dinitrotoluene, which were chosen due to their environmental importance.

## Methodology

Two DFT functionals M05-2X [14, 15] and B3LYP [16–18] expressed in 6-31+G(d,p) [19–23] basis set with aqueous solution modeled by IEFPCM continuum solvent model [24] utilizing the UFF [25] atom radii have been used. These levels of theory have been chosen based on our previous studies [26, 27]. Energy calculations for the selected stationary points have been carried out using the same functionals in combination with a significantly larger basis set, aug-cc-pVTZ [28]. Except for the hydrogen abstraction from the methyl group where unrestricted open shell method [29] was applied, singlet state using default restricted closed shell method was used. All quantum-mechanical calculations were performed using Gaussian package G09 rev. A.02 [30] with default convergence criteria. Vibrational analysis was performed not only to confirm that obtained optimized geometries indeed correspond to stationary points (either local minimum or first order saddle point) on the potential energy surfaces but also to evaluate contributions of vibrational motions to thermochemistry calculations. Merz-Singh-Kollman population analysis [31, 32] has been performed for all obtained stationary points. Transition states of modeled reactions have been located using Berny algorithm [33, 34]. All reaction pathways have been investigated using intrinsic reaction coordinate (IRC) [35] protocol in which end points have been subsequently optimized to either reactants or products. Calculations of reaction pathways probabilities, were based on Eyring-Polanyi equation [36–38]. The influence of the tunneling was tested using Wigner correction [39]. Aromaticity indexes have been calculated for all structures using reformulated harmonic oscillator model of aromaticity (HOMA) [40, 41]. Bond orders were calculated using Pauling equation [42].

## Results and discussion

### Toluene

**Fig. 1:**
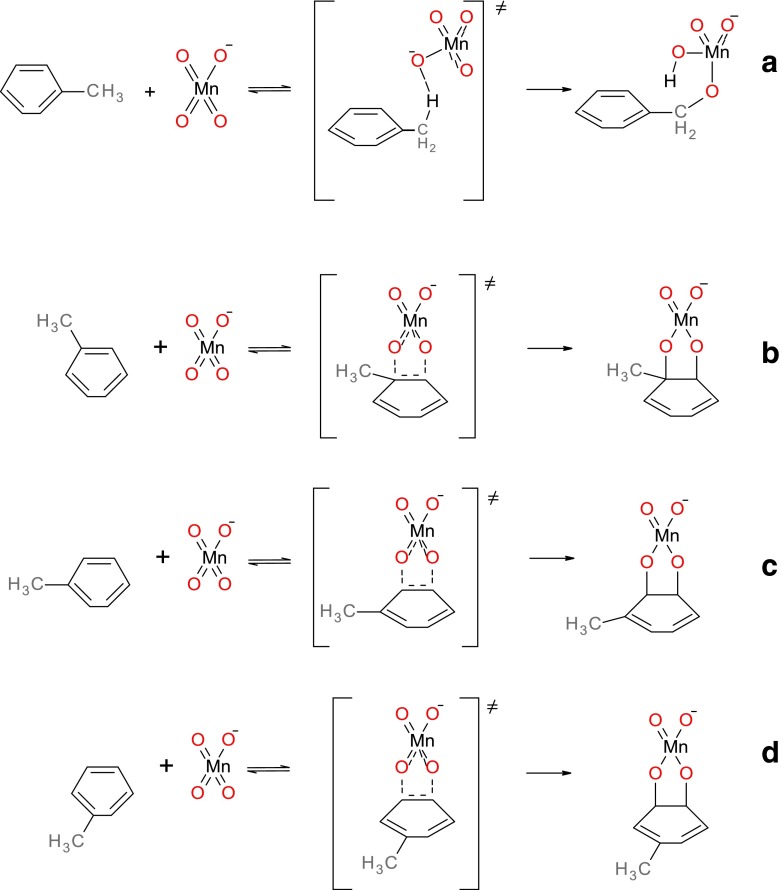
Possible reactions of toluene and with permanganate anion at positions: **a** C_m_, **b** C_1_-C_2_, **c** C_2_-C_3_, **d** C_3_-C_4_

Environmental studies of the oxidation of toluene nitroderivatives by permanganate anion show that hydrogen atom abstraction from the methyl group competes with the ring oxidation. The first, rate-determining step in the case of hydrogen abstraction is formation of the benzyl radical while in the case of the ring oxidation it is formation of the adduct. We have considered a simple model for these processes, i.e., reactions of permanganate anion with toluene using previously employed theory level [26, 27]. Three different regio-selective attacks of permanganate anion on the aromatic ring are possible here as presented in Fig. 1. Results collected in Table 1 show that Gibbs free energy of activation (ΔG^≠^) for aromatic ring oxidation is smaller than the one for the hydrogen atom abstraction from methyl group. The percentage contributions of alternative pathways of toluene oxidation, %F, (calculated from ratios of Gibbs free energies of activation) do not, however, agree with the experimental results [5], which indicate that the predominant pathway of this reaction is hydrogen atom abstraction from the methyl group. These results question the applicability of the theory level used previously [26, 27] to the present systems. We have, therefore, started our studies by identifying a theory level that properly describes the competition between the pathways a – d presented in Fig. 1.

**Table 1:** Activation Gibbs free energies, exothermicity and contribution of alternative pathways (%F) of oxidation of toluene by permanganate anion at several different theory levels (all with IEFPCM)

Attack	ΔG^≠^ [kcal mol^−1^]	ΔG_R_ [kcal mol^−1^]	% F
M05-2X/6-31+G(d,p)			
C_m_	25.0	−71.9	0.0 (0.0)
C_1_-C_2_^a^	21.0	−53.9	10.4 (10.4)
C_2_-C_3_	19.7	−54.2	86.1 (86.1)
C_3_-C_4_	21.6	−52.4	3.5 (3.5)
M05-2X/6-31+G(d,p) with ZPC and thermal corrections			
C_m_	22.2	−66.6	25.6
C_1_-C_2_	22.6	−48.4	12.8
C_2_-C_3_	21.8	−48.1	48.2
C_3_-C_4_	22.6	−47.0	13.3
M05-2X/aug-cc-pVTZ//M05-2X/6-31+G(d,p) with ZPC and thermal corrections			
C_m_	18.3	−70.2	99.8
C_1_-C_2_	22.5	−50.2	0.1
C_2_-C_3_	22.7	−50.3	0.1
C_3_-C_4_	26.1	−49.2	0.0
B3LYP/6-31+G(d,p)			
C_m_	25.9	−25.9	13.1 (16.5)
C_1_-C_2_	26.1	−5.8	10.2 (9.8)
C_2_-C_3_	25.2	−6.8	43.6 (41.9)
C_3_-C_4_	25.4	−6.4	33.1 (31.8)
B3LYP/6-31+G(d,p) with ZPC and thermal corrections			
C_m_	27.2	−20.2	99.7
C_1_-C_2_	32.2	2.4	0.1
C_2_-C_3_	30.8	0.9	0.2
C_3_-C_4_	33.3	3.4	0.0
B3LYP/aug-cc-pVTZ//B3LYP/6-31+G(d,p) with ZPC and thermal corrections			
C_m_	24.4	−21.0	99.9
C_1_-C_2_	33.3	4.0	0.0
C_2_-C_3_	32.0	2.6	0.1
C_3_-C_4_	34.6	5.0	0.0
Experimental^5^			
C_m_			100
Ring oxidation			0

^a^For atom numbering see Fig. 2

Since the percentage contribution of the competing pathways results from the energetic barriers, we have extended IEFPCM/M05-2X/6-31+G(d,p) by including ZPC and thermal corrections and by calculating energies using larger basis set. As can be seen from the results listed in Table 1, only after including all of these correction does one obtain the agreement between experiment and theory. When, however, B3LYP functional has been used instead of M05-2X, even the results obtained with smaller basis set became acceptable when ZPC and thermal corrections were included (see the last three entries in Table 1). Furthermore, inclusion of tunneling correction (values reported in parenthesis in the last column) did not affect the results significantly. Therefore B3LYP/6-31+G(d,p) with ZPC and thermal corrections has been used in the present studies.

**Fig. 2:**
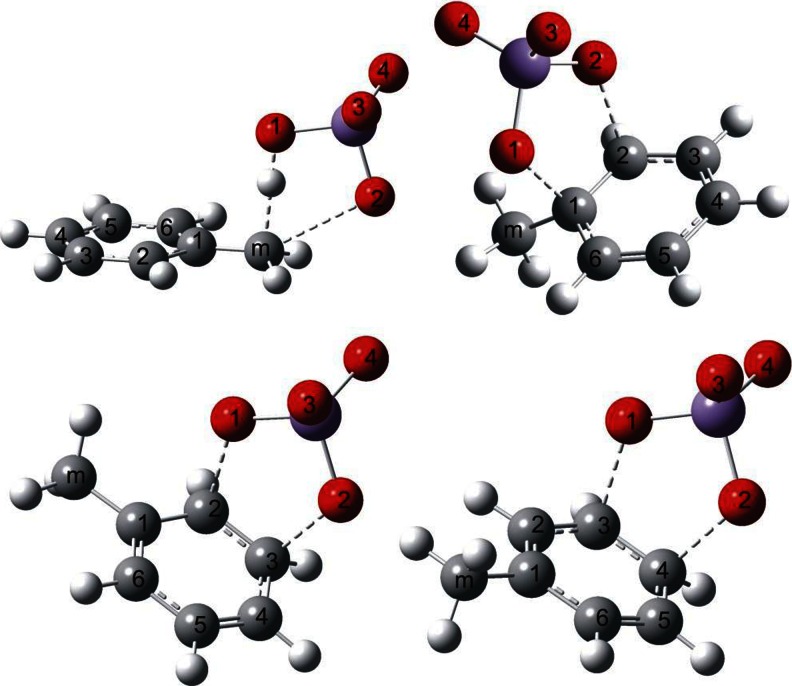
Optimized structures of transition states of modeled oxidation reactions of toluene with permanganate anion at positions (from *upper*, *left*): C_m_, C_1_-C_2_, C_2_-C_3_, and C_3_-C_4_

Optimized structures of the transition states corresponding to alternative pathways of toluene oxidation by permanganate together with atom numbering used are shown in Fig. 2. In all ring oxidation cases, we have observed formation of the C-O-Mn-O-C ring, which is almost perpendicular to the aromatic ring surface. In the case of methyl group oxidation, the atoms H-O-Mn-O-C form a similar pseudo-cyclic structure. No bridging structures of transition states corresponding to C_1_-C_3_, C_1_-C_4_ attacks or combining ring carbon with methyl group carbon attack have been observed; all these initial structures converged to one of those presented in Fig. 2.

Geometric results are presented in Table 2. In all reactions involving ring oxidation, similar products differing only in the position of attack, are obtained. Corresponding changes of bond distances are also almost identical in all three cases. The same is true for valence angles despite the fact that initial values differ significantly. These reactions proceed analogically to benzene oxidation [27] however transition state structures are not symmetric due to steric hindrance caused by the methyl group. In the transition state of toluene oxidation at the C_1_-C_2_ bond these distances are different and larger than in other cases; C_1_-O_1_ bond length equals to 1.98 Å and C_2_-O_2_ is 1.96 Å, corresponding to bond orders of 0.22 and 0.23, respectively. Elongation of these bonds is a consequence of the steric hindrance exerted by the methyl group. In reactions in which the attack occurs at C_2_-C_3_ and C_3_-C_4_ the corresponding values are 1.94 Å and 1.95 Å (bond order of about 0.24) and 1.97 Å (bond orders of 0.22), respectively. Interestingly, the above bond orders for toluene oxidation do not correlate with the barriers as one would expect a slightly earlier transition state for the reaction with lowest barrier. Dihedral angle Φ, defined as C-C-O-Mn, varies for all considered reactions indicating that permanganate anion rotates over the aromatic ring. Interestingly, in the case of both C_2_-C_3_ and C_3_-C_4_ oxidation, MnO_4_^-^ rotates in one direction, stops at the transition state (dihedral angles are almost 0), and then rotates back but in the case of C_1_-C_2_ attack rotation is in one direction only which again may be ascribed to the presence of steric hindrance.

**Table 2:** Selected geometric parameters (d – distances in Å, α,*Φ* – angles in °) and HOMA aromaticity indexes of modeled oxidation processes of toluene with permanganate ions (R – reactants, TS – transition states, P – products) at IEFPCM/B3LYP/6-31+G(d,p) theory level

Parameter	*R*	*TS*	*P*	Parameter	*R*	*TS*	*P*
Toluene C_m_ attack				Toluene C_1_-C_2_ attack			
* d*_*Cm-H*_	1.098	1.521	2.448	*d*_*C1-C2*_	1.403	1.451	1.552
* d*_*H-O1*_	2.583	1.103	0.969	*d*_*C1-O1*_	4.430	1.978	1.450
* d*_*Cm-O2*_	4.245	2.726	1.414	*d*_*C2-O2*_	4.564	1.957	1.435
* d*_*O1-Mn*_	1.600	1.703	1.819	*d*_*O1-Mn*_	1.600	1.658	1.804
* d*_*O2-Mn*_	1.600	1.629	1.822	*d*_*O2-Mn*_	1.600	1.661	1.806
* α*_*O1-H-Cm*_	169.5	179.3	116.8	*α*_*O1-C1-C2*_	109.0	103.9	106.7
* α*_*H-Cm-O2*_	35.5	54.6	80.2	*α*_*C1-C2-O2*_	86.7	106.6	107.9
* α*_*Cm-O2-Mn*_	94.3	103.8	126.4	*α*_*C2-O2-Mn*_	100.4	115.5	115.0
* α*_*O2-Mn-O1*_	109.4	95.5	97.9	*α*_*O2-Mn-O1*_	109.5	97.4	87.8
* α*_*Mn-O1-H*_	117.4	105.4	112.1	*α*_*Mn-O1-C1*_	94.8	116.1	115.3
* Φ*_*C1-Cm-O2-Mn*_	−89.1	3.3	−82.3	*Φ*_*C1-Cm-O2-Mn*_	29.5	−5.6	−23.7
* HOMA*	0.958	0.909	0.962	*HOMA*	0.959	0.459	−1.890
Toluene C_2_-C_3_ attack				Toluene C_3_-C_4_ attack			
* d*_*C2-C3*_	1.398	1.444	1.537	*d*_*C3-C4*_	1.398	1.444	1.538
* d*_*C2-O1*_	4.589	1.942	1.443	*d*_*C3-O1*_	4.730	1.969	1.437
* d*_*C3-O2*_	4.936	1.968	1.435	*d*_*C4-O2*_	4.586	1.945	1.443
* d*_*O1-Mn*_	1.600	1.662	1.807	*d*_*O1-Mn*_	1.600	1.661	1.808
* d*_*O2-Mn*_	1.600	1.660	1.810	*d*_*O2-Mn*_	1.600	1.662	1.807
* α*_*O1-C2-C3*_	102.4	105.8	107.4	*α*_*O1-C3-C4*_	78.1	105.0	107.3
* α*_*C2-C3-O2*_	92.1	105.4	107.1	*α*_*C3-C4-O2*_	115.0	106.2	107.9
* α*_*C3-O2-Mn*_	97.3	115.3	114.3	*α*_*C4-O2-Mn*_	110.0	115.8	113.9
* α*_*O2-Mn-O1*_	109.5	97.4	87.9	*α*_*O2-Mn-O1*_	109.4	97.4	80.0
* α*_*Mn-O1-C2*_	106.4	116.1	113.7	*α*_*Mn-O1-C3*_	123.4	115.5	114.8
* Φ*_*C1-Cm-O2-Mn*_	34.1	1.4	25.9	*Φ*_*C1-Cm-O2-Mn*_	−28.9	0.1	−25.2
* HOMA*	0.958	0.485	−1.722	*HOMA*	0.958	0.490	−1.701

We have carried out calculations of HOMA indices to compare how addition at different positions of the ring influences the aromaticity. Indices collected in Tables 2 and 3 indicate that dearomatization during all toluene ring oxidation reactions increases as the attack occurs closer to the methyl group. Interestingly, this trend is opposite to the one that could be expected from the C-O bond lengths in the corresponding transition state structures; the shortest being observed for C_2_-C_3_ (average of 1.55 Å) and the longest for C_1_-C_2_ attack (average of 1.97 Å). This result of the steric hindrance exerted by the neighboring methyl group illustrates how subtle the balance is between different factors influencing reactivity in the opposite directions.

**Table 3:** Bond lengths in aromatic rings (in Å) for HOMA analysis of modeled oxidation processes of toluene with permanganate ions (R – reactants, TS – transition states, P – products) at IEFPCM/B3LYP/6-31+G(d,p) theory level

Parameter	*R*	*TS*	*P*	Parameter	*R*	*TS*	*P*
Toluene C_m_ attack				Toluene C_1_-C_2_ attack			
C_1_-C_2_	1.405	1.417	1.403	C_1_-C_2_	1.403	1.451	1.552
C_2_-C_3_	1.398	1.393	1.398	C_2_-C_3_	1.399	1.438	1.508
C_3_-C_4_	1.399	1.402	1.399	C_3_-C_4_	1.398	1.369	1.344
C_4_-C_5_	1.398	1.402	1.398	C_4_-C_5_	1.399	1.434	1.466
C_5_-C_6_	1.399	1.393	1.399	C_5_-C_6_	1.398	1.368	1.345
C_1_-C_6_	1.404	1.417	1.403	C_1_-C_6_	1.405	1.445	1.515
* HOMA*	0.958	0.909	0.962	*HOMA*	0.959	0.459	−1.890
* EN*	0.040	0.066	0.037	*EN*	0.039	0.224	1.157
* GEO*	0.002	0.025	0.001	*GEO*	0.002	0.317	1.733
Toluene C_2_-C_3_ attack				Toluene C_3_-C_4_ attack			
C_1_-C_2_	1.404	1.449	1.519	C_1_-C_2_	1.404	1.373	1.347
C_2_-C_3_	1.398	1.444	1.537	C_2_-C_3_	1.399	1.438	1.509
C_3_-C_4_	1.399	1.437	1.508	C_3_-C_4_	1.398	1.444	1.538
C_4_-C_5_	1.399	1.369	1.345	C_4_-C_5_	1.399	1.440	1.509
C_5_-C_6_	1.399	1.434	1.467	C_5_-C_6_	1.398	1.368	1.345
C_1_-C_6_	1.404	1.372	1.349	C_1_-C_6_	1.405	1.442	1.475
* HOMA*	0.958	0.485	−1.722	*HOMA*	0.958	0.490	−1.701
* EN*	0.040	0.224	1.128	*EN*	0.040	0.224	1.117
* GEO*	0.002	0.291	1.594	*GEO*	0.002	0.286	1.584

The above differences regarding the reaction advancement in the transition state gathered from electronic and geometric data were further investigated by performing Merz-Singh-Kollman population analysis (see Table 4). The analysis revealed that initial charges in the reactant complex with orientation for the attack at the C_1_-C_2_ bond are 0.38 a.u. for C_1_ and −0.32 a.u. at C_2_. In the transition state the charge on C_1_ remains unchanged while that on C_2_ becomes positive (0.25 a.u.). In the other two reactions these charges systematically and simultaneously increase. In all cases attacked carbons become positively charged in product followed by increasing negative charge on both attacking oxygen atoms and reduction of positive charge on manganese atom.

**Table 4:** Charge distribution based on Merz-Singh-Kollman population analysis of selected atoms in modeled oxidation processes of toluene with permanganate ions (R – reactants, TS – transition states, P – products) at IEFPCM/B3LYP/6-31+G(d,p) theory level

Parameter	*R*	*TS*	*P*	Parameter	*R*	*TS*	*P*
Toluene C_m_				Toluene C_1_-C_2_ attack			
* H*	0.202	0.435	0.396	*C*_*1*_	0.383	0.381	0.782
* C*_*m*_	−0.628	−0.782	0.196	*C*_*2*_	−0.324	0.246	0.638
* O*_*1*_	−0.543	−0.708	−0.867	*O*_*1*_	−0.546	−0.567	−0.721
* O*_*2*_	−0.539	−0.632	−0.677	*O*_*2*_	−0.546	−0.572	−0.764
* Mn*	1.163	1.219	1.244	*Mn*	1.192	1.172	1.152
* O*_*3*_	−0.542	−0.619	−0.672	*O*_*3*_	−0.553	−0.646	−0.659
* O*_*4*_	−0.544	−0.619	−0.662	*O*_*4*_	−0.551	−0.668	−0.669
Toluene C_2_-C_3_ attack				Toluene C_3_-C_4_ attack			
* C*_*2*_	−0.320	−0.067	0.401	*C*_*3*_	−0.105	0.213	0.347
* C*_*3*_	−0.107	0.220	0.327	*C*_*4*_	−0.177	−0.054	0.67
* O*_*1*_	−0.543	−0.485	−0.635	*O*_*1*_	−0.555	−0.544	−0.675
* O*_*2*_	−0.546	−0.519	−0.671	*O*_*2*_	−0.555	−0.524	−0.680
* Mn*	1.183	1.110	1.150	*Mn*	1.226	1.176	1.108
* O*_*3*_	−0.543	−0.636	−0.660	*O*_*3*_	−0.560	−0.653	−0.650
* O*_*4*_	−0.552	−0.663	−0.671	*O*_*4*_	−0.559	−0.675	−0.661

In the reaction of hydrogen atom abstraction one of the oxygen atoms attacks the hydrogen atom of methyl group while another oxygen atoms moves in the direction of the methyl carbon (and in fact, in the subsequent step the C_m_-O bond is formed). The pseudo-cyclic H-O-Mn-O-C structure is nearly perpendicular to the aromatic ring. The geometry of this part of the transition state structure is similar to the one obtained with higher basis set [13] although breaking the toluene C-H bond at IEFPCM/B3LYP/6-31+G(d,p) has the length of about 1.52 Å (bond order of 0.30) while it is 1.67 Å in the case of B3LYP/6-311++G(d,p) while forming O-H bond is about 1.1 Å (bond order of 0.68) in the case of IEFPCM/B3LYP/6-31+G(d,p) and 1.05 Å in the case of higher basis set. A small difference is also observed in the forming C-O bond; 2.73 Å in the case of smaller basis set (bond order of 0.02) and 2.70 Å in the case of higher. Evolution of the dihedral angle throughout oxidation of toluene is quite interesting. These values change from −89.1° for reactants through 3.3° at the transition state to −82.3° for products.

Analysis of HOMA indices for the methyl group oxidation is also interesting; temporary lowering of aromaticity is observed in the transition state. Analysis of HOMA factors indicate that in this case bond elongation term (EN) is responsible for the change, as opposite to the ring oxidation reactions where it was caused by the bond alternation term (GEO). Population analysis reveals that in the case of methyl group oxidation environment has lower influence on the charge distribution then in the case of aromatic ring oxidation. The attacked carbon atoms become more negatively charged in the transition state (change of 0.15 a.u.) but in the products they are positively charged. Positive charge located initially on the abstracted hydrogen atom (about 0.20 a.u.) increases in the transition state (about 0.43 a.u.) and decreases in products (about 0.40 a.u.). These results do not support earlier suggestions of the hydride transfer in the toluene oxidation by permanganate [10].

### 2-Nitro- and 2,6-dinitrotoluene

**Fig. 3:**
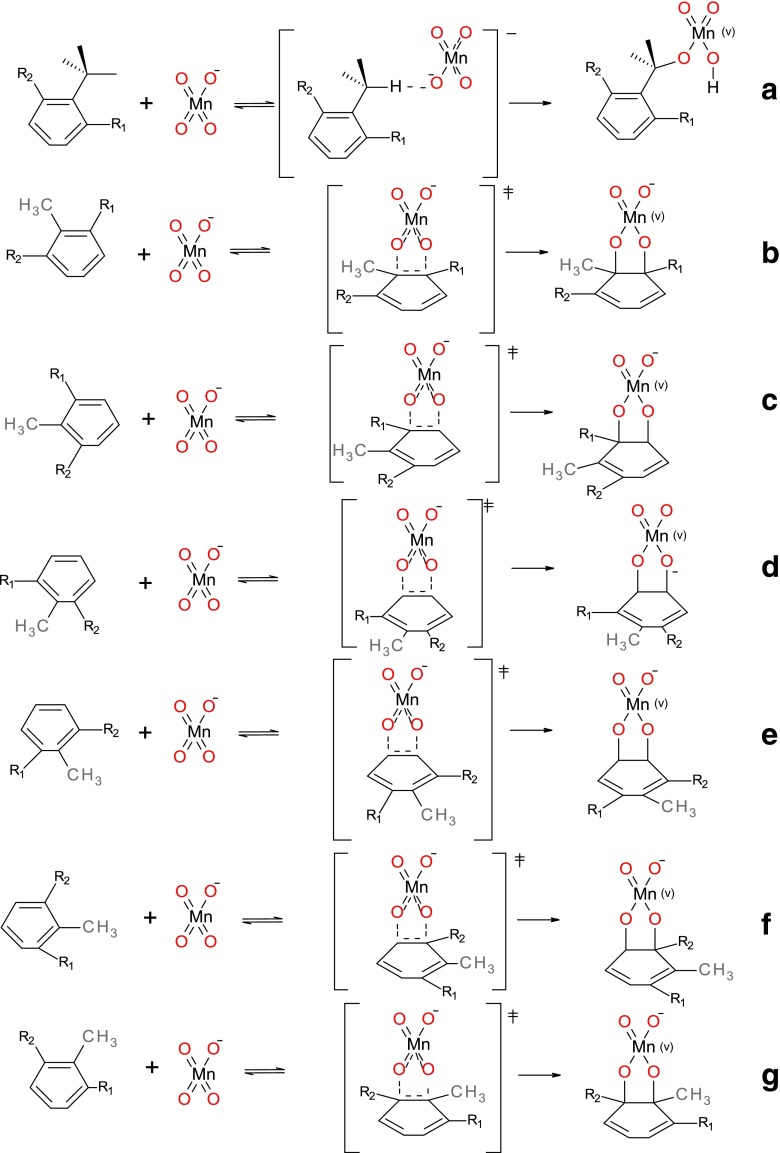
Modeled oxidation reactions of 2-nitrotoluene (R_1_ = NO_2_, R_2_ = H) and 2,6-dinitrotoluene (R_1_, R_2_ = NO_2_) with permanganate at positions: **a** C_m_, **b** C_1_-C_2_, **c** C_2_-C_3_, **d** C_3_-C_4_, **e** C_4_-C_5_, **f** C_5_-C_6_, **g** C_1_-C_6_

We have selected these two compounds as models because of the extreme differences in relative contributions of alternative oxidation pathways observed for them experimentally. While in the case of symmetrically substituted dinitroderivative alternative pathways are similar to those found for toluene the situation is more complicated in the case of monosubstitution since all six possible ring oxidation processes lead to different products; schematic representation of all possible pathways is given in Fig. 3. In Tables 5, 6, 7, 8, 9, 10 and 11 corresponding energetic parameters and resulting percentage contributions of each alternative reaction in the overall conversion of 2-nitrotoluene and 2,6-nitrotoluene are collected.

**Table 5:** Activation Gibbs free energies, exothermicity, contribution of alternative pathways and comparison of methyl group vs. ring oxidation with experimental data in oxidation reactions of 2-nitrotoluene and 2,6-dinitrotoluene by permanganate anion

Attack	ΔG^≠^ [kcal mol^−1^]	ΔG_R_ [kcal mol^−1^]	% F	% F_DFT_/% F_exp_^5^
2-nitrotoluene				
C_m_	25.8	−19.1	93.1	93/87
C_1_-C_2_	29.9	−5.0	0.1	7/13
C_2_-C_3_	28.5	−7.7	1.0	
C_3_-C_4_	27.9	−2.7	3.2	
C_4_-C_5_	28.9	0.2	0.6	
C_5_-C_6_	28.2	−0.7	1.8	
C_1_-C_6_	29.4	−0.4	0.2	
2,6-dinitrotoluene				
C_m_	24.2	−20.3	67.6	68/58
C_1_-C_2_	28.0	−8.3	0.1	32/42
C_2_-C_3_	26.1	−11.0	2.5	
C_3_-C_4_	24.7	−2.2	29.8

**Table 6:** Selected geometric parameters (d – distances in Å, α,*Φ* – angles in °) of modeled oxidation processes of 2,6-dinitrotoluene with permanganate ions (R – reactants, TS – transition states, P – products) at IEFPCM/B3LYP/6-31+G(d,p) theory level

Parameter	*R*	*TS*	*P*	Parameter	*R*	*TS*	*P*
2,6-dinitrotoluene C_m_ attack				2,6-dinitrotoluene C_1_-C_2_ attack			
* d*_*Cm-H*_	1.096	1.593	2.863	*d*_*C1-C2*_	1.409	1.475	1.582
* d*_*m-O1*_	2.434	1.077	0.966	*d*_*C1-O1*_	4.056	1.711	1.418
* d*_*Cm-O2*_	3.828	2.897	1.407	*d*_*C2-O2*_	4.325	2.400	1.369
* d*_*O1-Mn*_	1.601	1.701	1.814	*d*_*O1-Mn*_	1.599	1.679	1.817
* d*_*O2-Mn*_	1.599	1.604	1.822	*d*_*O2-Mn*_	1.599	1.609	1.844
* α*_*O1-H-Cm*_	169.4	176.5	116.9	*α*_*O1-C1-C2*_	76.7	107.3	105.5
* α*_*H-Cm-O2*_	44.1	55.1	60.8	*α*_*C1-C2-O2*_	119.1	100.1	109.5
* α*_*Cm-O2-Mn*_	98.3	100.4	141.7	*α*_*C2-O2-Mn*_	99.7	103.2	115.9
* α*_*O2-Mn-O1*_	109.2	99.3	101.2	*α*_*O2-Mn-O1*_	109.4	100.9	86.1
* α*_*Mn-O1-H*_	112.1	108.8	111.5	*α*_*Mn-O1-C1*_	134.3	127.2	141.8
* Φ*_*C1-Cm-O2-Mn*_	83.9	−22.9	−21.3	*Φ*_*C1-C2-O2-Mn*_	−12.4	9.3	17.6
2,6-dinitrotoluene C_2_-C_3_ attack				2,6-dinitrotoluene C_3_-C_4_ attack			
* d*_*C2-C3*_	1.396	1.457	1.562	*d*_*C3-C4*_	1.391	1.444	1.536
* d*_*C2-O1*_	3.857	2.314	1.363	*d*_*C3-O1*_	3.358	1.725	1.423
* d*_*C3-O2*_	3.315	1.678	1.427	*d*_*C4-O2*_	4.085	2.229	1.433
* d*_*O1-Mn*_	1.599	1.614	1.841	*d*_*O1-Mn*_	1.600	1.689	1.817
* d*_*O2-Mn*_	1.603	1.692	1.807	*d*_*O2-Mn*_	1.599	1.623	1.815
* α*_*O1-C2-C3*_	61.2	99.3	110.2	*α*_*O1-C3-C4*_	124.3	109.2	107.6
* α*_*C2-C3-O2*_	129.6	110.8	107.9	*α*_*C3-C4-O2*_	75.7	101.5	108.6
* α*_*C3-O2-Mn*_	104.6	124.5	115.5	*α*_*C4-O2-Mn*_	111.6	106.8	113.7
* α*_*O2-Mn-O1*_	108.9	99.9	86.8	*α*_*O2-Mn-O1*_	109.3	99.4	87.7
* α*_*Mn-O1-C2*_	116.9	105.4	116.4	*α*_*Mn-O1-C3*_	117.9	122.6	114.8
* Φ*_*C2-C3-O2-Mn*_	57.7	2.9	−18.5	*Φ*_*C3-C4-O2-Mn*_	11.8	−7.4	−24.5

**Table 7:** Charge distribution based on Merz-Singh-Kollman population analysis of selected atoms in modeled oxidation processes of 2,6-dinitrotoluene with permanganate ions (R – reactants, TS – transition states, P – products) at IEFPCM/B3LYP/6-31+G(d,p) theory level

Parameter	*R*	*TS*	*P*	Parameter	*R*	*TS*	*P*
2,6-dinitrotoluene C_m_ attack				2,6-dinitrotoluene C_1_-C_2_ attack			
* H*	0.254	0.403	0.373	*C*_*1*_	0.045	0.136	0.289
* C*_*m*_	−0.695	−0.745	−0.048	*C*_*2*_	0.104	0.330	0.941
* O*_*1*_	−0.532	−0.650	−0.820	*O*_*1*_	−0.539	−0.413	−0.618
* O*_*2*_	−0.532	−0.541	−0.587	*O*_*2*_	−0.539	−0.473	−0.711
* Mn*	1.148	1.242	1.201	*Mn*	1.186	1.087	1.103
* O*_*3*_	−0.543	−0.533	−0.647	*O*_*3*_	−0.552	−0.520	−0.585
* O*_*4*_	−0.536	−0.538	−0.640	*O*_*4*_	−0.551	−0.523	−0.589
2,6-dinitrotoluene C_2_-C_3_ attack				2,6-dinitrotoluene C_3_-C_4_ attack			
* C*_*2*_	0.070	0.071	0.293	*C*_*3*_	−0.302	−0.012	0.136
* C*_*3*_	−0.259	0.221	0.658	*C*_*4*_	0.023	−0.139	0.399
* O*_*1*_	−0.525	−0.477	−0.640	*O*_*1*_	−0.518	−0.450	−0.570
* O*_*2*_	−0.537	−0.479	−0.694	*O*_*2*_	−0.533	−0.462	−0.607
* Mn*	1.161	1.171	1.155	*Mn*	1.151	1.107	1.070
* O*_*3*_	−0.542	−0.538	−0.593	*O*_*3*_	−0.542	−0.542	−0.597
* O*_*4*_	−0.535	−0.546	−0.606	*O*_*4*_	−0.543	−0.555	−0.613

**Table 8:** Bond lengths in aromatic rings (in Å) for HOMA analysis of modeled oxidation processes of 2,6-dinitrotoluene with permanganate ions (R – reactants, TS – transition states, P – products) at IEFPCM/B3LYP/6-31+G(d,p) theory level

Parameter	*R*	*TS*	*P*	Parameter	*R*	*TS*	*P*
2,6-dinitrotoluene C_m_ attack				2,6-dinitrotoluene C_1_-C_2_ attack			
C_1_-C_2_	1.410	1.444	1.412	C_1_-C_2_	1.409	1.475	1.582
C_2_-C_3_	1.396	1.394	1.396	C_2_-C_3_	1.396	1.405	1.508
C_3_-C_4_	1.391	1.391	1.391	C_3_-C_4_	1.391	1.379	1.344
C_4_-C_5_	1.391	1.391	1.391	C_4_-C_5_	1.391	1.407	1.450
C_5_-C_6_	1.396	1.395	1.396	C_5_-C_6_	1.396	1.378	1.349
C_1_-C_6_	1.408	1.444	1.412	C_1_-C_6_	1.409	1.484	1.537
* HOMA*	0.956	0.726	0.944	*HOMA*	0.956	0.243	−2.502
* EN*	0.029	0.123	0.035	*EN*	0.029	0.286	1.398
* GEO*	0.015	0.151	0.021	*GEO*	0.015	0.470	2.104
2,6-dinitrotoluene C_2_-C_3_ attack				2,6-dinitrotoluene C_3_-C_4_ attack			
Parameter	*R*	*TS*	*P*	Parameter	*R*	*TS*	*P*
C_1_-C_2_	1.408	1.435	1.527	C_1_-C_2_	1.409	1.386	1.353
C_2_-C_3_	1.396	1.457	1.562	C_2_-C_3_	1.396	1.459	1.517
C_3_-C_4_	1.392	1.459	1.500	C_3_-C_4_	1.391	1.444	1.536
C_4_-C_5_	1.391	1.353	1.339	C_4_-C_5_	1.391	1.395	1.501
C_5_-C_6_	1.397	1.433	1.463	C_5_-C_6_	1.396	1.389	1.342
C_1_-C_6_	1.409	1.392	1.354	C_1_-C_6_	1.409	1.437	1.478
* HOMA*	0.957	0.344	−2.063	*HOMA*	0.956	0.543	−1.695
* EN*	0.030	0.289	1.245	*EN*	0.029	0.237	1.140
* GEO*	0.013	0.367	1.819	*GEO*	0.015	0.220	1.556

**Table 9:** Selected geometric parameters (d – distances in Å, α,*Φ* – angles in °) of modeled oxidation processes of 2-nitrotoluene with permanganate ions (R – reactants, TS – transition states, P – products) at IEFPCM/B3LYP/6-31+G(d,p) theory level

Parameter	*R*	*TS*	*P*	Parameter	*R*	*TS*	*P*
2-nitrotoluene C_m_ attack							
* d*_*Cm-H*_	1.095	1.538	2.504				
* d*_*H-O1*_	2.559	1.097	0.967				
* d*_*Cm-O2*_	4.066	2.862	1.412				
* d*_*O1-Mn*_	1.600	1.700	1.816				
* d*_*O2-Mn*_	1.599	1.615	1.824				
* α*_*O1-H-Cm*_	166.0	178.5	116.0				
* α*_*H-Cm-O2*_	46.6	54.4	81.6				
* α*_*Cm-O2-Mn*_	95.8	100.6	127.7				
* α*_*O2-Mn-O1*_	109.4	97.9	99.3				
* α*_*Mn-O1-H*_	117.1	107.9	115.3				
* Φ*_*C1-Cm-O2-O1*_	−85.0	−94.2	−81.2				
2-nitrotoluene C_1_-C_2_ attack				2-nitrotoluene C_2_-C_3_ attack			
* d*_*C1-C2*_	1.412	1.469	1.571	*d*_*C2-C3*_	1.401	1.455	1.549
* d*_*C1-O1*_	4.229	1.729	1.431	*d*_*C2-O1*_	3.597	2.234	1.368
* d*_*C2-O2*_	3.957	2.268	1.345	*d*_*C3-O2*_	4.376	1.718	1.427
* d*_*O1-Mn*_	1.599	1.685	1.808	*d*_*O1-Mn*_	1.599	1.627	1.844
* d*_*O2-Mn*_	1.599	1.623	1.838	*d*_*O2-Mn*_	1.599	1.688	1.810
* α*_*O1-C1-C2*_	73.2	108.3	106.3	*α*_*O1-C2-C3*_	118.2	100.5	110.2
* α*_*C1-C2-O2*_	122.4	100.9	109.6	*α*_*C2-C3-O2*_	80.1	110.1	107.4
* α*_*C2-O2-Mn*_	104.2	106.9	116.3	*α*_*C3-O2-Mn*_	108.3	122.9	115.1
* α*_*O2-Mn-O1*_	109.4	98..9	86.4	*α*_*O2-Mn-O1*_	109.4	98.9	86.8
* α*_*Mn-O1-C1*_	115.4	124.6	116.1	*α*_*Mn-O1-C2*_	121.8	107.6	115.3
* Φ*_*C1-C2-O2-O1*_	−8.7	4.8	15.3	*Φ*_*C2-C3-O2-O1*_	−8.3	1.6	21.4
2-nitrotoluene C_3_-C_4_ attack				2-Nitrotoluene C_4_-C_5_ attack			
* d*_*C3-C4*_	1.389	1.440	1.532	*d*_*C4-C5*_	1.399	1.447	1.534
* d*_*C3-O1*_	3.931	1.846	1.436	*d*_*C4-O1*_	4.397	2.099	1.425
* d*_*C4-O2*_	4.478	2.055	1.427	*d*_*C5-O2*_	4.347	1.788	1.439
* d*_*O1-Mn*_	1.599	1.671	1.813	*d*_*O1-Mn*_	1.599	1.641	1.819
* d*_*O2-Mn*_	1.599	1.645	1.817	*d*_*O2-Mn*_	1.600	1.68	1.813
* α*_*O1-C3-C4*_	121.0	106.9	107.1	*α*_*O1-C4-C5*_	109.9	103.3	107.9
* α*_*C3-C4-O2*_	121.7	104.6	106.9	*α*_*C4-C5-O2*_	86.2	107.9	107.4
* α*_*C4-O2-Mn*_	76.2	111.7	113.7	*α*_*C5-O2-Mn*_	119.5	120.1	113.4
* α*_*O2-Mn-O1*_	115.7	98.0	87.7	*α*_*O2-Mn-O1*_	109.5	98.1	87.9
* α*_*Mn-O1-C3*_	109.4	118.8	113.2	*α*_*Mn-O1-C1*_	105.1	110.3	113.8
* Φ*_*C3-C4-O2-O1*_	−1.5	−0.3	28.0	*Φ*_*C4-C5-O2-O1*_	16.5	−4.9	27.2
2-nitrotoluene C_5_-C_6_ attack				2-nitrotoluene C_1_-C_6_ attack			
* d*_*C5-C6*_	1.395	1.443	1.534	*d*_*C1-C6*_	1.405	1.459	1.562
* d*_*C5-O1*_	3.501	1.811	1.432	*d*_*C1-O1*_	3.774	1.833	1.437
* d*_*C6-O2*_	4.128	2.099	1.435	*d*_*C6-O2*_	4.375	2.087	1.426
* d*_*O1-Mn*_	1.601	1.676	1.811	*d*_*O1-Mn*_	1.599	1.671	1.810
* d*_*O2-Mn*_	1.600	1.640	1.816	*d*_*O2-Mn*_	1.600	1.642	1.813
* α*_*O1-C5-C6*_	132.9	107.8	106.9	*α*_*O1-C1-C6*_	124.1	105.4	106.3
* α*_*C5-C6-O2*_	58.6	103.2	108.0	*α*_*C1-C6-O2*_	73.4	105.1	107.1
* α*_*C6-O2-Mn*_	116.9	110.7	113.4	*α*_*C6-O2-Mn*_	115.0	110.6	114.5
* α*_*O2-Mn-O1*_	108.9	98.2	87.8	*α*_*O2-Mn-O1*_	109.4	98.1	87.4
* α*_*Mn-O1-C2*_	160.1	119.6	114.4	*α*_*Mn-O1-C1*_	115.0	120.7	115.1
* Φ*_*C5-C6-O2-O1*_	41.6	6.7	26.5	*Φ*_*C1-C6-O2-O1*_	−21.2	−0.2	−27.5

**Table 10:** Charge distribution based on Merz-Singh-Kollman population analysis of selected atoms in modeled oxidation processes of 2,6-dinitrotoluene with permanganate ions (R – reactants, TS – transition states, P – products) at IEFPCM/B3LYP/6-31+G(d,p) theory level

Parameter	*R*	*TS*	*P*	Parameter	*R*	*TS*	*P*
2-nitrotoluene C_m_ attack							
* H*	0.214	0.395	0.474				
* C*_*m*_	−0.585	−0.824	0.294				
* O*_*1*_	−0.538	−0.648	−0.915				
* O*_*2*_	−0.543	−0.574	−0.713				
* Mn*	1.175	1.212	1.285				
* O*_*3*_	−0.546	−0.567	−0.671				
* O*_*4*_	−0.548	−0.570	−0.674				
2-nitrotoluene C_1_-C_2_ attack				2-nitrotoluene C_2_-C_3_ attack			
* C*_*1*_	0.191	0.382	0.472	*C*_*2*_	0.055	0.136	0.638
* C*_*2*_	0.126	0.451	1.042	*C*_*3*_	−0.239	0.141	0.400
* O*_*1*_	−0.534	−0.524	−0.716	*O*_*1*_	−0.551	−0.495	−0.631
* O*_*2*_	−0.528	−0.529	−0.772	*O*_*2*_	−0.556	−0.485	−0.576
* Mn*	1.160	1.163	1.165	*Mn*	1.240	1.133	1.110
* O*_*3*_	−0.550	−0.582	−0.618	*O*_*3*_	−0.568	−0.570	−0.597
* O*_*4*_	−0.541	−0.590	−0.622	*O*_*4*_	−0.561	−0.585	−0.612
2-nitrotoluene C_3_-C_4_ attack				2-nitrotoluene C_4_-C_5_ attack			
* C*_*3*_	−0.271	0.063	0.408	*C*_*4*_	−0.142	−0.144	0.159
* C*_*4*_	−0.090	−0.063	0.187	*C*_*5*_	−0.065	0.319	0.634
* O*_*1*_	−0.516	−0.484	−0.620	*O*_*1*_	−0.540	−0.490	−0.626
* O*_*2*_	−0.531	−0.498	−0.640	*O*_*2*_	−0.540	−0.512	−0.662
* Mn*	1.129	1.137	1.130	*Mn*	1.176	1.121	1.074
* O*_*3*_	−0.534	−0.605	−0.638	*O*_*3*_	−0.545	−0.590	−0.616
* O*_*4*_	−0.540	−0.624	−0.642	*O*_*4*_	−0.549	−0.610	−0.628
2-nitrotoluene C_5_-C_6_ attack				2-nitrotoluene C_1_-C_6_ attack			
* C*_*5*_	−0.127	0.342	0.335	*C*_*1*_	0.234	0.440	0.780
* C*_*6*_	−0.193	−0.305	0.414	*C*_*6*_	−0.200	0.058	0.420
* O*_*1*_	−0.545	−0.508	−0.671	*O*_*1*_	−0.520	−0.537	−0.702
* O*_*2*_	−0.544	−0.474	−0.619	*O*_*2*_	−0.536	−0.528	−0.713
* Mn*	1.188	1.093	1.114	*Mn*	1.154	1.162	1.169
* O*_*3*_	−0.548	−0.579	−0.629	*O*_*3*_	−0.543	−0.604	−0.644
* O*_*4*_	−0.547	−0.601	−0.638	*O*_*4*_	−0.547	−0.626	−0.653

**Table 11:** Bond lengths in aromatic rings (in Å) for HOMA analysis of modeled oxidation processes of 2-nitrotoluene with permanganate ions (R – reactants, TS – transition states, P – products) at IEFPCM/B3LYP/6-31+G(d,p) theory level

Parameter	*R*	*TS*	*P*	Parameter	*R*	*TS*	*P*
2-nitrotoluene C_m_ attack							
C_1_-C_2_	1.412	1.432	1.406				
C_2_-C_3_	1.401	1.406	1.401				
C_3_-C_4_	1.389	1.385	1.390				
C_4_-C_5_	1.399	1.407	1.399				
C_5_-C_6_	1.395	1.383	1.394				
C_1_-C_6_	1.405	1.426	1.403				
* HOMA*	0.948	0.824	0.962				
* EN*	0.038	0.088	0.030				
* GEO*	0.014	0.088	0.008				
2-nitrotoluene C_1_-C_2_ attack				2-nitrotoluene C_2_-C_3_ attack			
C_1_-C_2_	1.412	1.469	1.571	C_1_-C_2_	1.412	1.438	1.525
C_2_-C_3_	1.401	1.422	1.509	C_2_-C_3_	1.401	1.455	1.549
C_3_-C_4_	1.389	1.369	1.343	C_3_-C_4_	1.389	1.457	1.506
C_4_-C_5_	1.399	1.431	1.464	C_4_-C_5_	1.399	1.361	1.343
C_5_-C_6_	1.395	1.360	1.342	C_5_-C_6_	1.395	1.430	1.464
C_1_-C_6_	1.405	1.473	1.519	C_1_-C_6_	1.405	1.379	1.348
* HOMA*	0.948	0.230	−2.230	*HOMA*	0.948	0.385	−1.921
* EN*	0.038	0.275	1.263	*EN*	0.038	0.264	1.186
* GEO*	0.014	0.495	1.967	*GEO*	0.014	0.351	1.735
2-nitrotoluene C_3_-C_4_ attack				2-nitrotoluene C_4_-C_5_ attack			
C_1_-C_2_	1.412	1.395	1.370	C_1_-C_2_	1.412	1.449	1.481
C_2_-C_3_	1.401	1.449	1.513	C_2_-C_3_	1.401	1.382	1.344
C_3_-C_4_	1.389	1.440	1.532	C_3_-C_4_	1.389	1.412	1.503
C_4_-C_5_	1.399	1.421	1.502	C_4_-C_5_	1.399	1.447	1.534
C_5_-C_6_	1.395	1.376	1.346	C_5_-C_6_	1.395	1.451	1.508
C_1_-C_6_	1.405	1.429	1.466	C_1_-C_6_	1.405	1.369	1.348
* HOMA*	0.948	0.597	−1.471	*HOMA*	0.948	0.478	−1.625
* EN*	0.038	0.237	1.151	*EN*	0.038	0.237	1.089
* GEO*	0.014	0.166	1.320	*GEO*	0.014	0.284	1.537
2-nitrotoluene C_5_-C_6_ attack				2-nitrotoluene C_1_-C_6_ attack			
C_1_-C_2_	1.412	1.395	1.359	C_1_-C_2_	1.412	1.467	1.527
C_2_-C_3_	1.401	1.436	1.467	C_2_-C_3_	1.401	1.382	1.354
C_3_-C_4_	1.389	1.359	1.340	C_3_-C_4_	1.389	1.411	1.448
C_4_-C_5_	1.399	1.449	1.504	C_4_-C_5_	1.399	1.379	1.347
C_5_-C_6_	1.395	1.443	1.534	C_5_-C_6_	1.395	1.416	1.503
C_1_-C_6_	1.405	1.429	1.527	C_1_-C_6_	1.405	1.459	1.562
* HOMA*	0.948	0.501	−1.726	*HOMA*	0.948	0.454	−1.975
* EN*	0.038	0.240	1.163	*EN*	0.038	0.248	1.221
* GEO*	0.014	0.259	1.564	*GEO*	0.014	0.298	1.754

As can be seen ring attack probability increases with the increase of nitro groups attached to the aromatic ring. In the case of 2-nitrotoluene the obtained activation Gibbs free energy of methyl group oxidation suggests that reaction proceeds almost exclusively through the methyl group oxidation (93 %). In the case of 2,6-dinitrotoluene oxidation the it is only around 68 % with the remaining 32 % proceeding mostly by the attack at the C_3_-C_4_ bond. This is probably caused by the electron-withdrawing properties of this substituent, which may have negative influence on stabilization of the transition state of methyl group oxidation.

In the case of ring oxidation of nitroaromatics geometries of transition states differ significantly from those obtained for benzene [27] and toluene. Opposite to both C-O bonds (of about 1.95 Å) being nearly equally advanced in the transition state, in the case of toluene nitroderivatives these bonds are quite different; one of them oscillates around 1.7 Å corresponding to bond being nearly half formed (bond order of about 0.45), while the C-O distance remains quite large, around 2.2 Å indicating that the formation of this bond hardly started (bond order of about 0.1). This asymmetry is smaller in the case of 2-nitrotoluene and diminishes slightly with the distance from the nitrosubstituent, with C-O forming bond lengths being about 1.8 and 2.1 Å. This is paralleled by significantly stronger dearomatization occurring in the transition states of ring oxidation of 2-nitro- and 2,6-dinitrotoluene for the attack involving C_1_-C_2_ and C_2_-C_3_ bonds. Charge distribution on attacking oxygen atoms follows the same pattern in all reactions of initial slight increase from about −0.55 a.u. to −0.48 a.u. on the transition from the reactants to the transition state and final decrease in the products to average of −0.65 a.u. With the sole exception of the unusually small partial charge on the C_6_ atom (−0.31 a.u.) in the reaction proceeding with the attack on the C_5_-C_6_ bond, atomic charges on the attacked carbon atoms, on the other hand, generally increase systematically from the reactant complex to the transition state to product although absolute changes between reactions are very diverse.

**Fig. 4:**
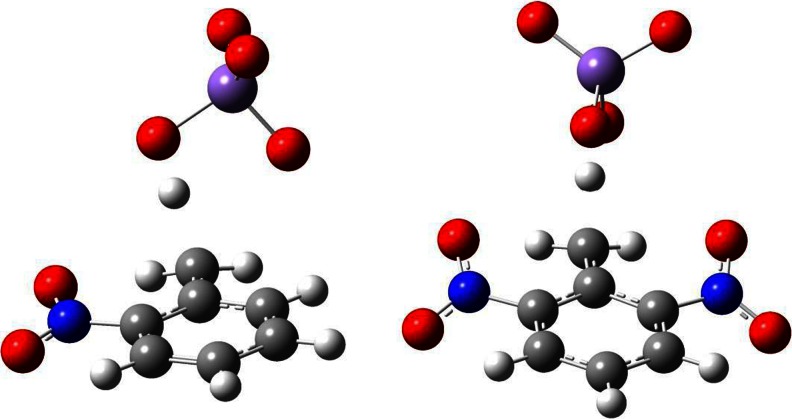
Transition state structures of methyl group oxidation in modeled oxidation reactions of 2-nitrotoluene and 2,6-dinitrotoluene with permanganate

As illustrated in Fig. 4 geometries of the transition states of the methyl group oxidation of the considered nitroderivatives are significantly different. In the case of 2,6-dinitrotoluene the structure is almost symmetric and very similar to the one observed in the corresponding toluene oxidation. In the case of 2-nitrotoluene, however, the permanganate anion is rotated about 90 degrees relative to the C_m_-C_1_ bond. Changes of the dihedral angle Φ throughout the 2,6-dinitrotoluene oxidation molecule are similar to those observed in the case of toluene. In the case of 2-nitrotolune, however, these changes are negligible; the dihedral angle changes from −85° in reactants complex to −94° in the transition state to −81° in the product.

The length of the breaking C-H bond in 2,6-dinitrotoluene transition state is 1.59 Å, which corresponds to the bond order of 0.24, and is longer than in the case of mono-nitrosubstituted derivative where the corresponding values to 1.54 Å and 0.29, respectively. Analogously, the forming O-H bond in the doubly substituted derivative transition state is 1.08 Å (bond order of 0.73) and is noticeably shorter than in the case of 2-nitrotoluene where the corresponding values are 1.10 Å and 0.69. These results indicate that in both reactions the transition states are late. The overall trend obtained in our studies shows, in agreement with expectations, the increasingly later transition state in the order: toluene, 2-nitrotoluene, 2,6-nitrotoluene. This sequence agrees with the calculated ring dearomatization in the transition state, which increases from toluene to 2-nitrotoluene and 2,6-nitrotoluene with the corresponding HOMA indices equal to 0.91, 0.82 and 0.73, respectively. Partial atomic charges of reacting C · · · H · · · O atoms, on the other hand, do not reveal any significant differences; all changes follow the same pattern although the absolute values differ.

## Conclusions

We have performed calculations of alternative oxidation pathways of toluene and its *ortho*-substituted nitroderivatives by permanganate anion. Based on the obtained structures of reactants and transition states kinetic isotope effects for each carbon and nitrogen position and subsequently averaged elemental isotopic fractionation have been calculated. These values, compared with experimentally determined ones [5], validated the used theory level. This combination of theoretical and experimental analysis greatly enhances our understanding of oxidative degradation processes of common environmentally important aromatic pollutants.

Our studies show that the preference of the attack position of permanganate anion in oxidation reactions with selected aromatic compounds changes with positions and number of substituents in aromatic ring. On the example of the well studied [8–12] case of toluene oxidation we have shown that the correct preference of methyl group oxidation is predicted when Gibbs free energies from IEFPCM/B3LYP/6-31+G(d,p) calculations, including ZPC and thermal corrections, are used. Furthermore, applying continuum solvent model results in slightly earlier transition states than in the corresponding reaction modeled in gas phase [13]. Obtained charge distribution does not support hydride transfer in toluene oxidation by permanganate. For nitrosubstituted derivatives competitive ring oxidation has been predicted in agreement with the experiment.

From the chemical point of view, ring oxidation reactions proceed via relatively early transition states that become quite unsymmetrical for reactions involving *ortho*-nitrosubstituted derivatives. Transition states for the hydrogen atom abstraction reactions, on the other hand, are late, with C-H bond breaking advanced in about 70 %. All favored reactions are characterized by the Gibbs free energy of activation of about 25 kcal mol^−1^. Methyl group oxidations are exothermic by about 20 kcal mol^−1^ while ring oxidations are around thermoneutrality.
